# EZH2 represses mesenchymal genes and upholds the epithelial state of breast carcinoma cells

**DOI:** 10.1038/s41419-024-07011-y

**Published:** 2024-08-22

**Authors:** Amador Gallardo, Lourdes López-Onieva, Efres Belmonte-Reche, Iván Fernández-Rengel, Andrea Serrano-Prados, Aldara Molina, Antonio Sánchez-Pozo, David Landeira

**Affiliations:** 1 Centre for Genomics and Oncological Research (GENYO), Avenida de la Ilustración 114, 18016 Granada, Spain; 2 Department of Biochemistry and Molecular Biology II, Faculty of Pharmacy, University of Granada, Granada, Spain; 3 Instituto de Investigación Biosanitaria ibs.GRANADA, Hospital Virgen de las Nieves, Granada, Spain; 4 Department of Biochemistry and Molecular Biology I, Faculty of Science, University of Granada, Granada, Spain

**Keywords:** Breast cancer, Chromatin

## Abstract

Emerging studies support that the polycomb repressive complex 2 (PRC2) regulates phenotypic changes of carcinoma cells by modulating their shifts among metastable states within the epithelial and mesenchymal spectrum. This new role of PRC2 in cancer has been recently proposed to stem from the ability of its catalytic subunit EZH2 to bind and modulate the transcription of mesenchymal genes during epithelial-mesenchymal transition (EMT) in lung cancer cells. Here, we asked whether this mechanism is conserved in other types of carcinomas. By combining TGF-β-mediated reversible induction of epithelial to mesenchymal transition and inhibition of EZH2 methyltransferase activity, we demonstrate that EZH2 represses a large set of mesenchymal genes and favours the residence of breast cancer cells towards the more epithelial spectrum during EMT. In agreement, analysis of human patient samples supports that EZH2 is required to efficiently repress mesenchymal genes in breast cancer tumours. Our results indicate that PRC2 operates through similar mechanisms in breast and lung cancer cells. We propose that PRC2-mediated direct transcriptional modulation of the mesenchymal gene expression programme is a conserved molecular mechanism underlying cell dissemination across human carcinomas.

## Introduction

Polycomb group (PcG) proteins are hallmark epigenetic regulators of embryo development, stem cell differentiation and cancer [1–3]. PcG proteins associate to form multimeric complexes termed polycomb repressive complexes 1 and 2 (PRC1 and PRC2) that can post-translationally modify histone tails and repress gene transcription. RING1A/B is the catalytic subunit of PRC1 and monoubiquitinates lysine 119 on histone H2A (H2AK119ub). Likewise, EZH1/2 harbours the catalytic activity of PRC2 and trimethylates lysine 27 on histone H3 (H3K27me3). The coordinated activity of PRCs leads to the formation of chromatin domains enriched in H2AK119ub and H3K27me3 that facilitate transcriptional repression of hundreds of genes across the genome in a cell-type-specific manner. In stem cells, the activity of PRCs leads to the transcriptional repression of hundreds of lineage-inappropriate genes, contributing to maintaining a specific gene expression programme and defining stem cell identity [1–3].

In the context of cancer, initial studies revealed that PcG proteins act as oncogenes through the transcriptional repression of the *INK4A/ARF* (*CDKN2A*) tumour suppressor locus [4, 5]. However, subsequent studies confirmed that this was just one aspect of the role of PRC in cancer, because different PRC subunits can have senescence-independent prooncogenic activity or tumour suppressor function [2, 6]. In fitting with its role as a regulator of cell identity in stem cell biology, recent studies support that PRC2 can regulate dynamic phenotypic changes of cancer cells by modulating their transition between metastable states within the epithelial and mesenchymal spectrum through poorly understood mechanisms [6–9]. Reversible transition between the epithelial and mesenchymal states vertebrate carcinoma cell dissemination [10], and inhibitors of the PRC2 catalytic subunits EZH2 are currently being developed to treat several types of carcinomas as main or adjuvant therapy [11, 12]. Therefore, understanding the molecular basis of the function of PRC2 during EMT is crucial for a precise comprehension of cancer dissemination, and for successful application of PRC2 inhibitors in the clinics.

In breast cancer, comprehensive evidence indicates that EZH2 facilitates metastasis [13–16], but the underlying molecular mechanism is unclear. While initial reports suggested that EZH2 impacts metastasis progression through non-canonical pathways independent of EZH2 methyltransferase activity (i.e. p38 signalling and integrin B1-FAK) [13, 14], later studies supported that the role of EZH2 in breast cancer is based on the H3K27me3-mediated transcriptional repression of key candidate genes (ie. FOXC1 or GATA3) [15, 16]. Notably, a recent study hints at the interesting possibility that PRC2 regulates breast cancer metastasis by modulating the EMT process [9]. This finding is in consonance with evidence obtained in lung carcinoma cells, where the loss of function of EZH2 leads to the acquisition of mesenchymal features and changes in tumour colonisation capacity [6–9, 17]. Importantly, in lung carcinoma cells, the regulation of EMT by EZH2 is mediated by the ability of EZH2 to directly bind the gene promoter regions and co-ordinately modulate the transcription of many mesenchymal-associated genes through H3K27me3 [8]. Here, we asked whether direct transcriptional regulation of mesenchymal genes by EZH2 also occurs in breast carcinoma cells. We found that EZH2 represses a large set of mesenchymal genes and promotes the residence of breast cancer cells in a more epithelial state. We propose that direct transcriptional modulation of the mesenchymal gene expression programme by PRC2 is a conserved molecular mechanism across different types of carcinomas that contributes to equipping cells with the plasticity required for efficient cell dissemination.

## Results

### EZH2 represses mesenchymal genes in breast carcinoma cells

**Fig. 1:**
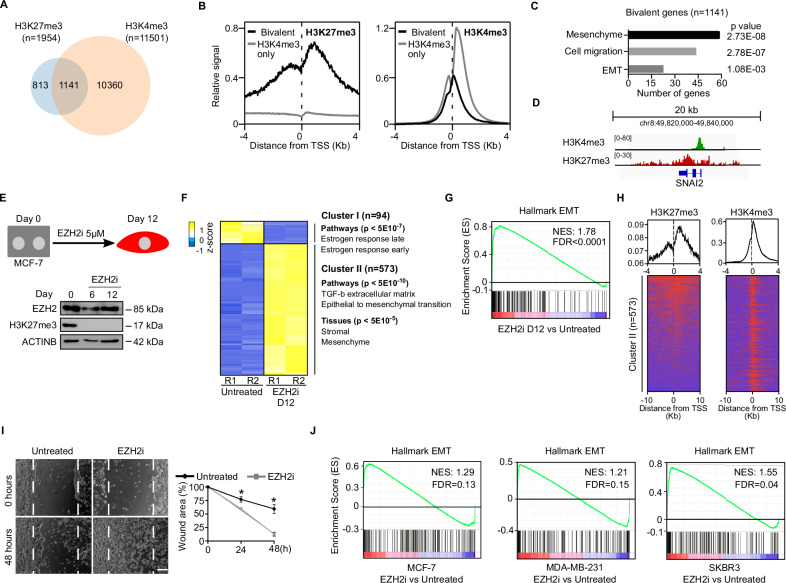
EZH2 represses mesenchymal genes in breast carcinoma cells. **A** Venn diagram comparing sets of gene promoters displaying enrichment of H3K27me3 or H3K4me3 at their promoter regions by ChIP-seq in MCF-7 cells. **B** Plots showing the ChIP-seq average binding profile of H3K27me3 and H3K4me3 around the TSS of gene promoters identified in (**A**) as bivalent (H3K27me3 + H3K4me3) or H3K4m3-only. **C** Gene Ontology analysis of bivalent genes identified in (**A**). **D** Genome browser view of H3K4me3 and H3K27me3 binding profiles at the *SNAI2* locus in MCF-7 cells. **E** Schematic diagram of the treatment of MCF-7 cells with EZH2i (upper panel). The lower panel shows western blot analysis of whole-cell extracts comparing the levels of EZH2 and H3K27me3 during the experiment. ACTIN B provides a loading control. **F** Heatmap analysis of mRNA expression of 667 differentially expressed genes upon 12 days of EZH2i treatment (FC > 4, *p* < 0.05) by RNA-seq of two independent replicates (R1 and R2) in MCF-7 cells. **G** GSEA of EMT associated genes in MCF-7 cells treated compared to untreated with EZH2i for 12 days. Normalised Enrichment Score (NES) and false discovery rate (FDR) are indicated. **H** Plots showing H3K27me3 and H3K4me3 enrichment measured by ChIP-seq around the TSS of the promoters of 573 genes identified in cluster II in Fig. 1F. **I** Brightfield images and quantification of cultured wound healing assays using MCF-7 cells that have been treated or not with EZH2i for four days. The mean and SEM of 3 experiments are shown. Asterisks indicate statistical significance using a Mann–Whitney test (* *p* < 0.05). **J** GSEA of EMT associated genes in MCF-7, MDA-MB-231 and SKBR3 cells plated at low density and treated with EZH2i for 14 days, compared to untreated controls. Normalised enrichment score (NES) and statistically significant false discovery rate (FDR < 0.25) are indicated.

To analyse the function of EZH2 in breast cancer, we focused on the human MCF-7 cell line because it is a well-established system to study the molecular basis of metastasis in breast adenocarcinomas. These cells are homozygous null mutants for the *CDKN2A* locus, which makes them a good model to study the *CDKN2A*-independent function of EZH2 in cancer. We first set to identify what genes are directly regulated by EZH2 in MCF-7 cells. EZH2 catalyses H3K27me3 at the promoter regions of target genes, and thus, the genome-wide distribution of H3K27me3 is a surrogate measure of EZH2 binding [1, 18]. Analysis of H3K27me3 enrichment maps revealed that EZH2 target the promoter region of 1954 genes in MCF-7 cells (Fig. 1A, Table S1). In pluripotent cells, many H3K27me3-repressed target genes display chromatin features of active transcription, such as trimethylation of H3K4 (H3K4me3) and they are usually referred to as bivalent genes [19]. Bivalent chromatin seems to facilitate their transcriptional activation during lineage transition [19]. Interestingly, a comparison of H3K27me3 and H3K4me3 genome-wide binding maps in MCF-7 cells revealed that 1141 genes (58.4%) out of the 1954 genes marked by H3K27me3 displayed a bivalent state, and hence they accumulated both modifications at their promoter region (Fig. 1A, B, Table S1). Importantly, bivalent genes were enriched for genes involved in the regulation of mesenchymal features, EMT and cell migration and included genes that are widely used as mesenchymal markers such as *N-CADHERIN* and *SNAI2* (Figs. 1C, D and S1A). Thus, we concluded that in MCF-7 cells, EZH2 binds a large set of mesenchymal genes that display features of bivalent chromatin. We hypothesised that this bivalent state might facilitate the coordinated transcriptional activation of mesenchymal genes in response to signalling molecules during EMT.

To address whether EZH2 represses the mesenchymal gene expression programme through H3K27me3 in breast cancer cells we treated MCF-7 cells with a highly specific small molecule that inhibits EZH2 methyltransferase activity (GSK126, thereafter referred to as EZH2i) [20] and analyse whether the loss of H3K27me3 induced the activation of the mesenchymal gene expression programme. Inhibition of EZH2 led to a drastic reduction of global levels of H3K27me3 without sensibly affecting EZH2 protein stability (Fig. 1E) and produced only mild inhibition of cell proliferation that did not impair long-term culture of MCF-7 cells (Fig. S1B). Importantly, reduced levels of H3K27me3 during 12 days of culture led to a robust transcriptional activation of the mesenchymal marker *N-CADHERIN* (Fig. S1C), which is a H3K27me3-positive direct target of EZH2 (Fig. S1A). Likewise, analysis of the expression of master EMT transcription factors (EMT-TFs) revealed an evident specific activation of the *SNAI2* gene (Figs. S1D, E and S1F), which is also enriched for H3K27me3 and H3K4me3 at its promoter region (Fig. 1D). As expected, activation of mesenchymal genes upon EZH2 inhibition was associated to the downregulation of the epithelial marker *E-CADHERIN* (Fig. S1G, H). Importantly, transcriptome profiling analysis by mRNA sequencing (mRNA-seq) demonstrated that treatment of MCF-7 cells with EZH2i induced a global reorganisation of the transcriptional programme that involved the upregulation of 573 genes enriched in EMT pathways (cluster II in Fig. 1F) and mesenchymal markers (Fig. 1G). As expected, many of these genes displayed H3K27me3 and H3K4me3 at their promoter regions (Fig. 1H). In addition, we noticed that genes involved in oestrogen response were downregulated upon EZH2i treatment (Fig. 1F, cluster I), suggesting that activation of EMT induces the inactivation of the oestrogen pathway signalling in MCF-7 cells. Importantly, activation of mesenchymal genes in MCF-7 cells upon depletion of H3K27me3 was functionally relevant because cells treated with EZH2i displayed obvious increased mobility compared to untreated cells in cultured wound healing assay (Fig. 1I). Thus, we concluded that EZH2 targets and represses the transcription of a large set of bivalent mesenchymal genes in the breast cancer cell line MCF-7.

To confirm that mesenchymal genes are transcriptionally induced upon EZH2i treatment we exposed MCF-7 cells to another highly specific inhibitor of EZH2 that is approved for cancer treatment by the US Food and Drug Administration (EPZ-6438, tazemetostat) [21], and measure its impact on gene expression by mRNA-seq. In keeping with our findings using EZH2i, treatment with EPZ-6438 inhibited H3K27me3 deposition (Fig. S2A), induced the transcriptional activation of EZH2i-responsive genes (Fig. S2B) and activated the mesenchymal gene expression programme (Fig. S2C). To discard that the effects observed in EZH2i-treated cells were due to off-target effects of the EZH2 inhibitors assayed we measured the expression of mesenchymal genes upon shRNA-mediated knockdown of EZH2 protein in MCF-7 cells (shEZH2 cells). As expected, shEZH2 cells showed reduced global H3K27me3 (Fig. S2D, E), transcriptional induction of mesenchymal genes (Fig. S2F) and increased protein levels of the mesenchymal TF SNAI2 (Fig. S2G, H).

To confirm that EZH2 functions as a gatekeeper of epithelial identity in breast carcinoma cells we analysed whether inhibition of EZH2 led to the transcriptional activation of mesenchymal genes in cell lines derived from different types of metastatic breast adenocarcinomas: MCF-7 cells (ER+/PR±/Her2−), SKBR3 cells (ER−, PR−, Her2+) and MDA-MB-231 cells (ER−/PR−/Her2−). Cells were treated with EZH2i for 4 days and then plated at low density to form colonies for fourteen extra days in the presence of EZH2i. Inhibition of EZH2 induced minor effects on cell growth (Fig. S2I), confirming the suitability of these cell lines to study the proliferation-independent role of EZH2. Notably, inhibition of EZH2 activity induced the expression of mesenchymal genes (Figs. 1J and S2J) that were H3K27me3-positive prior to EZH2 inhibition (Fig. S2K). The precise set of EMT genes induced varied in the three analysed cell lines (Fig. S2M), in fitting with previous observations indicating EMT can be induced through different combinations of EMT genes [22]. Consistently, different combinations of mesenchymal TFs were activated in the three different cell lines (Fig. S1L). Of note, *SNAI2* was gradually upregulated after 12 days of EZH2i treatment in exponentially growing MCF-7 cells (Fig. S1D, compare day six and day twelve). After 18 days of EZH2i treatment, *SNAI1*, *TWSIT1* and *ZEB1* were also over-expressed. This suggests that the persistent absence of H3K27me3 facilitates a time-dependent gradual activation of the EMT programme and transition into the mesenchymal state.

Overall, we concluded that H3K27me3 deposition through EZH2 is required to maintain the transcriptional repression of mesenchymal genes and favour the residence of breast carcinoma cells in an epithelial state.

### TGF-β-induced EMT is reversible in breast carcinoma cells

**Fig. 2:**
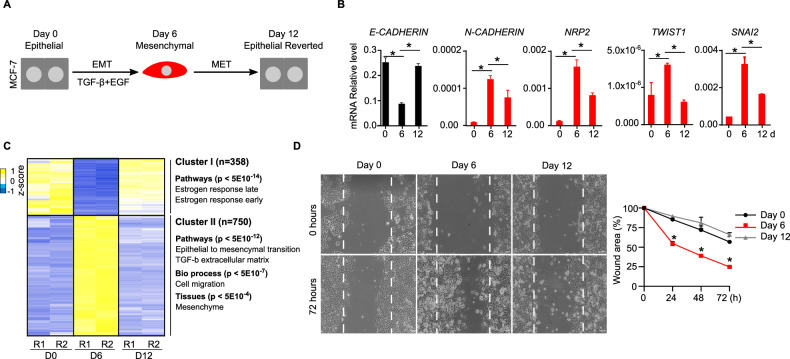
TGF-β-induced EMT is reversible in MCF-7 cells. **A** Scheme of the experimental design used to induce EMT-MET in MCF-7 cells. **B** Analysis of mRNA expression by RT-qPCR of indicated epithelial (black) or mesenchymal (red) genes during EMT-MET. Relative expression level against *GAPDH* and *ACTIN B* is shown. **C** Heatmap showing mRNA expression of 1108 genes that were reversibly regulated (FC > 2, *p* < 0.05) during EMT-MET. Two independent replicates (R1 and R2) at day 0 (E), day 6 (M) and day 12 (ER) are shown. Gene ontology analyses of genes in indicated clusters are shown. **D** Brightfield images and quantification plot comparing the wound healing capacity after 72 h of cells obtained at day 0, 6 and 12 during EMT–MET. The mean and SEM of 3 experiments are shown in (**B**) and (**D**). Asterisks indicate statistical significance using a Mann–Whitney test (* *p* < 0.05).

To examine whether EZH2 regulates transitions between the epithelial and mesenchymal states of breast cancer cells we setup an in vitro system to study the dynamics of EMT and its reverse process (mesenchymal to epithelial transition, MET) (Fig. 2A). Treatment of MCF-7 cells with 10 ng/ml transforming growth factor beta (TGF-β) and 50 ng/ml epidermal growth factor (EGF) during six days induced the transcription of mesenchymal markers (*N-CADHERIN, NRP2, TWIST1 and SNAI2*) and the downregulation of the epithelial marker *E-CADHERIN* (Fig. 2B). Withdrawal of TGF-β and EGF from the culture media during six additional days led to the reversion of transcriptional changes: downregulation of the expression of mesenchymal genes (*N-CADHERIN, NRP2, TWIST1 and SNAI2*) coupled to activation of the epithelial marker E-CADHERIN (Fig. 2B). Importantly, mRNA-seq analyses demonstrated that TGF-β stimulation induced a wide reversible reorganisation of the transcriptome that involved 1108 genes (Fig. 2C). This included the reversible activation of 750 genes enriched in EMT, cell migration and mesenchyme (cluster II, Fig. 2C), as well as 358 reversibly repressed genes that included factors involved in oestrogen receptor signalling (cluster I, Fig. 2C). Importantly, changes in the expression of epithelial and mesenchymal genes were accompanied by expected functional changes in cell mobility in cultured wound healing assays (Fig. 2D). We concluded that transient stimulation with TGF-β and EGF is a valid system to study EMT-MET in vitro in MCF-7 cells.

### EZH2 is required to repress mesenchymal genes during TGF-β-dependent MET in breast carcinoma cells

**Fig. 3:**
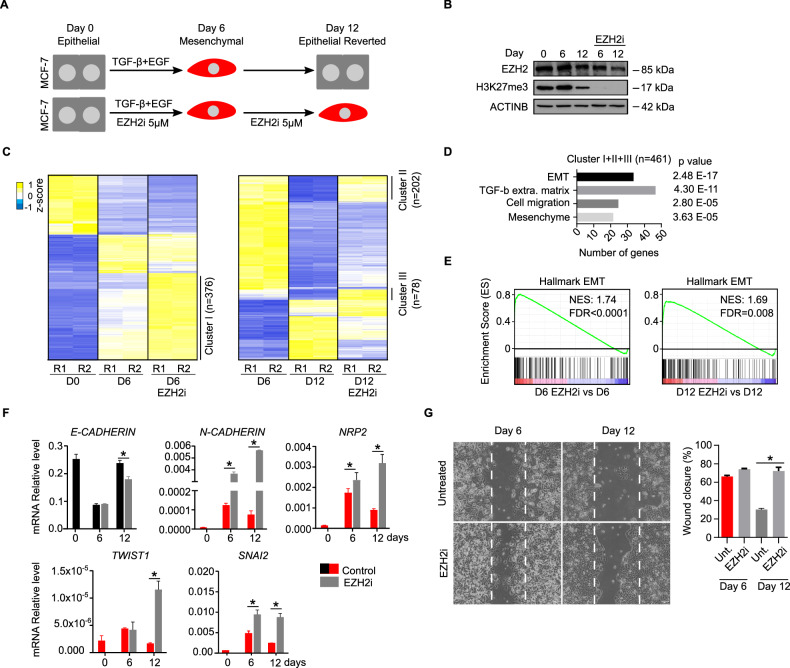
EZH2 is required to repress mesenchymal genes during TGF-β-dependent MET in MCF-7 cells. **A** Schematic diagram of the experimental conditions used to study the inhibition of EZH2 activity (EZH2i 5 µM) during reversible EMT–MET (TGF-β + EGF) in MCF-7 cells. **B** Western blot analysis of whole-cell extracts comparing the levels of EZH2 and H3K27me3 during the EMT-MET experiment described in (**A**). ACTIN B provides a loading control. **C** Heatmap showing the expression of 1108 reversible genes (identified in Fig. 2C) during EMT–MET in two biological replicates (R1 and R2) at day 0, day 6 (upon EMT) and day 12 (upon MET), in the presence or absence of EZH2i, are shown. Genes that are differentially expressed due to the presence of EZH2i are labelled as clusters I, II and III. **D** Gene ontology analysis of genes identified in clusters I, II and III in (**C**). **E** GSEA of EMT-associated genes in cells treated with EZH2i, relative to untreated, at day 6 (left) or day 12 (right). Normalised enrichment score (NES) and statistically significant false discovery rate (FDR < 0.25) are indicated. **F** RT-qPCR analysis showing mRNA level of indicated epithelial (black) and mesenchymal (red) genes during EMT–MET in the absence or presence of EZH2i. Expression level is calculated relative to *GAPDH* and *ACTIN B*. **G** Brightfield images and histogram analysing the effect of EZH2i treatment in wound healing closure after 72 h, in cells corresponding to day 6 or 12 during the EMT–MET described in (**A**). The mean and SEM of 3 experiments are shown in (**F**) and (**G**). Asterisks indicate statistical significance using a Mann–Whitney test (* *p* < 0.05).

To test whether inhibition of EZH2 alters EMT–MET we compared the behaviour of the 1108 reversible genes identified in Fig. 2C during transient TGF-β stimulation in the presence or absence of EZH2i and H3K27me3 marking (Fig. 3A, B). Inhibition of EZH2 enhanced the transcriptional induction of 376 genes during EMT (cluster I, Fig. 3C), and hindered the repression of 280 genes during MET (cluster II and cluster III, Fig. 3C). This set of 461 genes was highly enriched for genes involved in EMT, TGF-ß stimulation, cell migration and mesenchyme (Fig. 3D), indicating that EZH2 methyltransferase activity is required for reorganisation of epithelial-mesenchymal gene expression programmes during EMT-MET. In consonance, gene set enrichment analysis (GSEA) confirmed that MCF-7 cells treated with EZH2i displayed higher expression of mesenchymal genes than untreated cells (Fig. 3E). Likewise, analysis of the expression of epithelial (*E-CADHERIN*) and mesenchymal (*N-CADHERIN, NRP2, TWIST1 and SNAI2*) genes in cells treated or untreated with EZH2i also supported that inhibition of EZH2 promotes activation of mesenchymal markers during EMT and hinders their repression during MET (Fig. 3F). Expectedly, examination of the in vitro wound healing capacity of MCF-7 cells showed that treatment with EZH2i slightly increased the mobility of cells after six days of EMT (day six) and hindered the restoration of the more immobile epithelial phenotype upon MET (day twelve) (Fig. 3G). Therefore, we settled that EZH2 is required to downmodulate the expression of mesenchymal genes during EMT–MET in breast cancer cells and to allow efficient restoration of the epithelial state during MET.

### Expression of *EZH2* inversely correlates with the expression of mesenchymal genes in human breast tumours

**Fig. 4:**
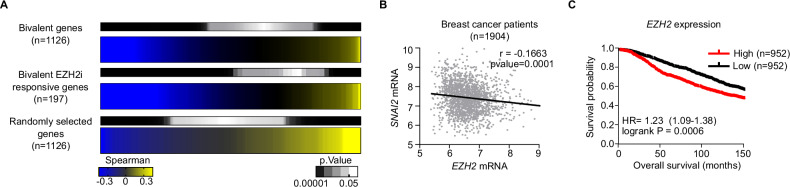
EZH2 level inversely correlates with the expression of mesenchymal genes, and it is associated with poor prognosis in breast cancer patients. **A** Heatmaps of Spearman´s correlation between the mRNA of *EZH2* and different subsets of genes in 1904 samples from breast cancer tumours. Bivalent genes include 1126 bivalent genes that were identified in Fig. 1A. Bivalent EZH2i responsive genes include 197 genes that are H3K27me3/H3K4me3-positive and are included in cluster II in Fig. 1F. The pattern of a set of 1126 randomly selected genes is shown for comparison purposes. **B** Spearman´s correlation of *EZH2* and *SNAI2* mRNA in 1904 patients of breast cancer. Each dot represents the expression values of one breast tumour sample. **C** Kaplan–Meier plots showing survival probability of 1904 breast cancer patients depending on the level of expression of *EZH2* mRNA.

To examine whether EZH2 represses mesenchymal genes in breast cancer cells in vivo, we used genome-wide gene expression datasets of 1904 resected breast cancer tumours available at the Molecular Taxonomy of Breast Cancer International Consortium. Importantly, we found that the levels of *EZH2* mRNA inversely correlate with the expression of EZH2 target genes (1126 out of the 1141 genes identified in Fig. 1A were analysed in these datasets) (Fig. 4A). Negative correlation was more accused for the group of 197 bivalent genes that were induced upon treatment with EZH2i (Fig. 4A). As expected, no significant correlation was found for a group of randomly selected control genes (Fig. 4A). The expression values of individual mesenchymal genes such as *SNAI2* also displayed the expected negative correlation (Fig. 4B). Therefore, these analyses support that EZH2 functions as a transcriptional repressor of the mesenchymal gene expression programme in human breast cancer tumours. In agreement with previous reports [23, 24], high expression of *EZH2* was associated with poor survival probability in our patient cohort (median survival, high: 132.3 months, low: 172.9 months) (Fig. 4C). Overall, we concluded that augmented expression of EZH2 is associated to reduced expression of EZH2-target mesenchymal genes and poor survival in breast cancer patients.

## Discussion

Metastasis causes around 90% of cancer-associated mortality, and therefore, understanding the mechanisms underlying metastatic dissemination is crucial to developing more effective therapies for cancer [10]. Metastatic dissemination relies on dynamic changes in carcinoma cell state that occur during epithelial to mesenchymal reversible transitions, and therefore, EMT–MET has emerged as a key druggable pathway in cancer intervention [10, 25]. Our study reveals that the PRC2 catalytic subunit EZH2 coordinates the repression of the mesenchymal gene expression programme in breast cancer cells, facilitating MET upon TGF-β stimulation decay. Because MET is required for efficient tumour colonisation [10, 25], our findings provide an explanation as to why EZH2-deficient breast cancer cells display reduced capacity to colonise new organs and form metastasis in mice models and patient-derived xenografts [13–16], as well as to the oncogenic behaviour of *EZH2* as a marker of poor prognosis in breast cancer patients (this study and [24, 26, 27]). Importantly, our discovery that EZH2 coordinates EMT–MET is in consonance with previous reports studying lung cancer cells where it has been shown that the loss of function of EZH2 hinders repression of mesenchymal genes during MET [8], and reduces tumour colonisation capacity in mouse models [6–8]. Counterintuitively, it has been recently reported that the loss of function of a non-catalytic PRC2 subunit (EED) in experimentally transformed human mammary epithelial cells (HMLER) leads to increased metastasis in mouse xenograft experiments [9]. This apparent discrepancy might rely on variations of EZH2 activity in the different loss of function systems: while in our experiments and previous reports in breast [15, 16] and lung [6–8] cancer the function of EZH2 was assayed in systems in which EZH2 protein level was reduced to undetectable levels, EED-depleted HMLER cells display only partial downregulation of EZH2 protein [9]. We propose that low activity of EZH2 protein in EED-depleted HMLER cells promotes the transition of breast cancer cells into a metastable mesenchymal state without fully impairing MET. This might explain the enhanced tumour colonisation capacity observed in EED mutant cells because the latest reports indicate that cancer malignancy and disease progression rely on the ability of cancer cells to reside in intermediate metastable states within the epithelial–mesenchymal spectrum rather than in extreme epithelial or mesenchymal states [28–30]. Overall, this study establishes a molecular framework that brings together previous reports in breast [9, 13–16] and lung [6–8] cancer cells, and supports a cell-of-origin-independent role of PRC2 as a direct modulator of the mesenchymal gene expression programme during EMT–MET in human carcinomas.

## Methods

### Breast cancer cell line culture conditions

The MCF-7, MDA-MB-231 and SKBR3 cell lines were kindly provided by the labs of Mª Jose Serrano and Dr. Juan Antonio Marchal (University of Granada, Spain). Cells were grown at 5% CO_2_ and 37 °C in DMEM high glucose media supplemented with 10% heat-inactivated foetal bovine serum (FBS) (Gibco), penicillin/streptomycin (Gibco), l-glutamine (Gibco) and 2-mercaptoethanol (Gibco). Detailed information about the cell lines used is provided in Table S2.

### Induction of in vitro EMT–MET by TGF-β and EGF stimulation in MCF-7 cells

Epithelial MCF-7 cells were plated at a density of 10,000 cells/cm^2^ and treated with 10 ng/mL TGF-β (Prepotech) and 50 ng/mL EGF (Prepotech) for 6 days to induce transition into a mesenchymal state (EMT). Thereafter, both cytokines were removed from the culture media and cells were grown for 6 additional days to allow reversion to the epithelial state (MET). Cells were trypsinized, counted and replated at initial density in fresh media every 48 h.

### Treatment of breast cancer cell lines with EZH2 inhibitors

EZH2 was inhibited using 5 µM GSK126 (A3446, APExBIO). Treatments with EZH2 inhibitor for 12 days were carried out by refreshing GSK126 every 2 days as MCF-7 cells were split to allow exponential cell growth. Likewise, during EMT–MET experiments, GSK126 was refreshed every two days as cells were diluted to the corresponding density (10,000 cells/cm^2^). In colony-forming assays, cells were pre-treated with GSK126 for 4 days to reduce the global H3K27me3 level before plating the cells. MCF-7, SKBR3 and MDA-MB-231 cells were seeded at a density of 200 cells/cm^2^ to allow the formation of colonies after 14 days. Media was refreshed every 5 days including GSK126 in the treated culture. In growth curves, cell viability and wound healing assays, cells were pre-treated with GSK126 for 4 days to reduce global H3K27me3 levels before the start of the experiment. Cells were maintained in the presence of GSK126 during the experiment as detailed below.

In experiments where EZH2 was inhibited using EPZ-6438, MCF-7 cells were exposed to 2 µM of EPZ-6438 (S7128, Deltaclon) for 12 days, and the inhibitor was refreshed every two days as MCF-7 were split to allow exponential cell growth.

### Knockdown of *EZH2* using lentiviral shRNA

pLKO.1 Puro plasmid containing a shRNA sequence against EZH2 (5′- CCCAACATAGATGGACCAAT-3′) was purchased in MERCK (TRCN000040077). Lentiviral particles were generated as previously described in [8]. Briefly, HEK293T cells were lipotransfected with pLKO.1 Puro shRNA-EZH2 (MERCK) together with packaging (psPAX2, Addgene plasmids #12260) and envelope (pMD2.G, Addgene #12259) plasmids (18 µg total DNA; plasmid proportions of 3:2:1, respectively). To generate a control line of lentiviral particles the same protocol was performed using a plasmid with no shRNA expression (pLKO.1 Puro, Addgene#8453). The transfection mixture was removed after 6 h incubation, and 8 ml of total medium was added. The viral supernatants were collected at 48 and 72 h, and filtered through a 0.45 mm filter (Cat#FPE404030, JET Biofil, Guangzhou, China), aliquoted and immediately frozen at −80 °C. Transduction of MCF-7 cells was carried out in three cycles on infection using 2 mL of the supernatant containing lentiviral particles of sh*EZH2* (pLKO.1 Puro shRNA-*EZH2*) or control (pLKO.1 Puro) respectively, and 8 µg/mL polybrene in 6x multiwells. sh*EZH2* and control MCF-7 cells were selected with 0.75 µg/mL of puromycin for 4 days. After the selection time, the cells were maintained in culture additionally for 12 days before performing the experiments to allow them to acquire mesenchymal features.

### Growth curve, cell viability and in vitro wound healing assays

To perform growth curves, cells were plated at a density of 10,000 cells/cm^2^ and diluted before confluence to the initial density. Accumulative growth was calculated by applying the dilution factor used. Inhibition of EZH2 was carried out by pre-treating cells with GSK126 for four days and refreshing the inhibitor in every cell dilution.

To analyse cell survival cells were pre-treated with GSK126 for 4 days, plated in 96-well plate at a density of 3000 cells per well. After 48 h, cell media was replaced by fresh media containing 0.1 mM of resazurin. The plate was incubated for 4 h at 37 °C and fluorescence at 585 nm wavelength was measured. Same procedure was applied to GSK126 untreated control cells. Survival was estimated as the ratio of the signal measured in treated cells relative to untreated control.

In cultured wound healing assays MCF-7 untreated or pre-treated for 4 days with GKS126 were grown to 100% confluence. A 1000 µL sterile pipette tip was used to produce a scratch in the monolayer of cells. Standard cell media was changed to media containing 1% of FBS and supplemented or not with GSK126. Cells were allowed to close the wound for 48 h. The area of the wound was imaged every 24 h using a widefield microscope and quantified using Image J software. The area of the wound at each time point was normalised to the area of the wound at time zero.

### RT-qPCR

RNA was isolated using Trizol reagent (Thermofisher), reverse transcribed using RevertAid Frist Strand cDNA synthesis kit (Thermofisher) and analysed by SYBRG real-time PCR using GoTaq qPCR Master Mix (Promega). Primers used are provided in supplementary Table S2.

### Western blot

Western blots of whole cell extracts, or histone preparations were carried out using standard procedures as previously described [31] (uncropped versions are presented in the original data file 1). The following primary antibodies were used: rabbit anti-EZH2 (Diagenode), mouse anti-H3K27me3 (Active Motif), rabbit anti-SNAI2 (CST), mouse anti-E-CADHERIN (BD), mouse anti-ACTIN B (Sigma-Aldrich), rabbit anti-ACTIN B (Cell signalling). Secondary species-specific antibodies conjugated to horseradish peroxidase were used: anti-rabbit-HRP (GE-Healthcare), anti-mouse-HRP (GE-Healthcare) and anti-goat-HRP (Abcam). Clarity Western ECL reagents (Bio-Rad) were used for detection. More information about the antibodies used is provided in Table S2.

### Immunofluorescence

Immunofluorescence analysis of MCF-7 cells was carried out as described previously [8]. Briefly, cells were fixed for 20 min in 2% paraformaldehyde, permeabilized for 5 min using 0.4% Triton-X100 and blocked for 30 min in phosphate buffer saline supplemented with 10% goat serum, 0.05% Tween 20 and 2.5% bovine serum albumin. A primary antibody against SNAI2 (CST) was used. The secondary antibody was anti-rabbit Alexa fluor 555 (Thermofisher). Slides were imaged by widefield fluorescence microscope Zeiss Axio Imager and images were analysed using Image J.

### ChIP sequencing analysis

Public ChIP-seq datasets of H3K27me3 and H3K4me3 performed in MCF-7, MDA-MB-231 and SKBR3 (Table S2) were analysed as follows. Alignment of the sequence reads was done using Bowtie2 [32], and human genome hg19 was used for mapping. Unmapped and multimapped reads were filtered out with SAMTools [33] and SamBamba [34] to keep only uniquely aligned sequences. BigWigs were generated after normalising with their input signal using the BamCompare function of the deepTools suite package [35]. Peak calling was performed with MACS version 3 [36] using the input as background for normalisation. Peaks with q ≤ 0.05 values were considered significant. Significant peaks were annotated with Homer software [37] by defining promoter regions as ±2 kb from the start of the transcription start site (TSS). The coverage of the samples around the TSS was performed with the Bioconductor package coverageView for a genomic window of ±4 kb using a bin size of 10 bp. R version 4.2.2 and RStudio version 2022.7.1.554 were used. BigWigs were generated using the deepTools suite [38] and reads per million (RPM) were used to represent the ChIP-seq signal.

### mRNA sequencing analysis

Total RNA was isolated using Trizol reagent (Thermofisher) or Rnease mini kit (Quiagen). Libraries and sequencing were performed at BGI Genomics. Strand-specific mRNA-seq libraries were generated using 200 ng of total RNA and the DNBSEQ library construction protocol. Libraries were sequenced using DNBSEQ high-throughput platform sequencing technology. Twenty million (150 bp paired-end) reads were obtained for each condition.

RNA sequencing data was analysed using the miARma-Seq pipeline [39]. First, quality control of reads was performed using FastQC software [40]. Reads were aligned using STAR 2.5.3a against the reference human genome hg19 (GENCODE assembly GRCh37.p13). To obtain expression values featureCounts 2.0.6 [41] was used. Reference gene annotations were obtained from the GENCODE assembly mentioned above. Normalisation of gene expression values was obtained by applying the trimmed mean values method (TMM) [42] using the NOISeq package [43]. Differential gene expression analysis was performed using the DESeq2 package [44]. GSEA [45] was performed against the set “Hallkmark Epithelial to Mesenchymal Transition” from the Molecular Signatures Database (MSigDB) [46].

### Bioinformatic analysis of breast cancer tumour samples

Information of 1904 breast cancer tumour samples from the Breast Cancer METABRIC dataset was analysed using CBioportal tools. Kaplan Meier survival analysis and the correlation analyses between mRNA levels of EZH2 of selected target genes were performed.

### Statistical analyses

Statistical significance (*p* < 0.05) was determined by applying a two-tailed non-parametric Mann–Whitney test. Spearman’s correlation coefficient was calculated to measure correlation among variables. All analyses were performed with GraphPad Prism 9 and/or R or Rstudio.

### Data access

Datasets are available at GEO-NCBI with accession number GSE247138.

### Supplementary information

Supplementary figures and table legends.

Figure S1

Figure S2

Table S2

Table S1

original western blots images
