# Supplementary material for: EZH2 represses mesenchymal genes and upholds the epithelial state of breast carcinoma cells
Source: Cell Death Dis. 2024 Aug 22;15(8):609. doi: 10.1038/s41419-024-07011-y (PMC11341823; doi:10.1038/s41419-024-07011-y)
Supplement: Supplementary file 1 — Supplementary figures and table legends. [file 41419_2024_7011_MOESM1_ESM.docx]

**Fig. S1. Treatment with EZH2i induces the activation of mesenchymal genes in MCF-7 cells.**

**(A)** Table showing bivalent genes classified related to mesenchyme identified in Fig. 1C.

**(B)** Growth curve of MCF-7 cells treated with EZH2i and untreated control cells.

**(C)** Analysis of *N-CADHERIN* mRNA expression by RT-qPCR during EZH2i treatment in MCF-7cells. Expression is calculated relative to housekeeping genes *GAPDH* and *ACTINB*.

**(D)** Analysis of EMT-TFs mRNA expression by RT-qPCR during EZH2i treatment in MCF-7cells. Expression is calculated relative to housekeeping genes *GAPDH* and *ACTINB*.

**(E)** Western blot analysis of SNAI2 in whole-cell extracts from EZH2i-treated MCF-7 cells. ACTIN B was used as a loading control.

**(F)** Immunofluorescence analysis of SNAI2 expression in MCF-7 cells treated with EZH2i. DAPI stains nuclear DNA. Scale bar represents 10 µm. Graph shows the quantification of the signal of SNAI2 in forty nuclei. Horizontal bar represents the median of the distribution.

**(G)** Histogram show the level of *E-CADHERIN* mRNA expression measured by RT-qPCR during EZH2i treatment in MCF-7 cells. Expression is calculated relative to housekeeping genes *GAPDH* and *ACTINB*.

**(H)** Western blot analysis of the level of E-CADHERIN in whole-cell extracts from EZH2i-treated MCF-7 cells. ACTIN B was used as a loading control.

Mean and SEM of 3 experiments are shown in B, C, D, G. Asterisks indicate statistical significance using a Mann-Whitney test (* p<0.05, **p<0.01).

**Fig. S2. Depletion of EZH2 activity induces the activation of mesenchymal genes in breast carcinoma cells.**

**(A)** Western blot analysis of whole-cell extracts comparing the levels of EZH2 and H3K27me3 during treatment of MCF-7 cells with 2 µM EPZ-6438 for 12 days. ACTINB provides a loading control.

**(B)** Boxplot comparing mRNA expression of 573 genes responsive to EZH2 inhibition (cluster II in Fig. 1F) by RNA-seq in MCF-7 cells treated or untreated for twelve days with EZH2 inhibitors GSK126 and EPZ-6438. Asterisks indicate statistical significance using a Mann-Whitney test (*** p<0.001).

**(C)** GSEA of EMT associated genes in MCF-7 cells treated with EZH2 inhibitor EPZ-6438 for 12 days. Normalized Enrichment Score (NES) and statistically significant false discovery rate (FDR<0.25) are indicated

**(D)** RT-qPCR analysis of the level of *EZH2* mRNA in MCF-7 cells expressing shRNA against *EZH2*.

**(E)** Western blot analysis of whole-cell extracts comparing the levels of EZH2 and H3K27me3 in shEZH2 and control MCF-7 cells. ACTINB provides loading control.

**(F)** RT-qPCR analysis showing the expression of mesenchymal genes in shEZH2 relative to control MCF-7 cells.

**(G)** Immunofluorescence analysis of SNAI2 expression in shEZH2 and control MCF-7 cells. DAPI stains nuclear DNA. Scale bar represents 10 µm.

**(H)** Graph showing the quantification of SNAI2 immunofluorescence (G) in thirty nuclei. Horizontal bar represents the median of the distribution.

**(I)** Histogram showing cell survival of indicated cells lines treated with EZH2i for six days relative to untreated control after resazurin staining and fluorescence quantification.

**(J)** Histogram showing the number of genes downregulated (grey bar) or upregulated (black bar) (FC > 2, p < 0.05) in indicated cell lines after plating cells at low density in the presence of EZH2i for 14 days. Terms enriched in gene ontology analyses are shown.

**(K)** Average enrichment ChIP-seq signal of H3K27me3 and H3K4me3 around the TSS of genes induced upon treatment with EZH2i in indicated cell lines.

**(L)** Histograms showing the level of mRNA expression of mesenchymal TFs in indicated cell lines treated or untreated with EZH2i conditions for 18 days.

**(M)** Venn diagram comparing sets of EMT genes induced after treatment with EZH2i for 14 days.

Mean and SEM of 3 experiments are shown in D, F and L. Asterisks indicate statistical significance using a Mann-Whitney test (* p<0.05, ***p<0.001).

**Supplementary Table S1.** List of genes used in genome-wide analyses.

**Supplementary Table S2.** List of reagents and public datasets.
