# Supplementary material for: EZH2 represses mesenchymal genes and upholds the epithelial state of breast carcinoma cells
Source: Cell Death Dis. 2024 Aug 22;15(8):609. doi: 10.1038/s41419-024-07011-y (PMC11341823; doi:10.1038/s41419-024-07011-y)

A

Process	Genes
**Cell surface**	*ALCAM;ALK;BMPR1B;CDH1;CXCR4;EPCAM;FLT1;GJA1;KIT;N-CADHERIN;NCAM;NGFR;PDGRA*
**Anti-cell adhesion**	*TNC*
**Growth factor**	*FGF9;FGF13*
**Cytokine**	*PTHLH*
**Ligand**	*BMP4;BMP6;BMP7;GDNF;WIF1;WNT7B*
**Cytoskeleton**	*CALD1*
**Extracellular matrix**	*FN1;FRAS1;HAS2*
**Transcription factor**	*DLX3;DLX5;ESR1;FOXC1;GLI1;HOXD11;NEUROG3;PGR;SNAI1;SNAI2;SOX5;TWIST1*
**Transcription regulation**	*BMI1;CITED1;ESRP1;EYA1;PRRX1*

B

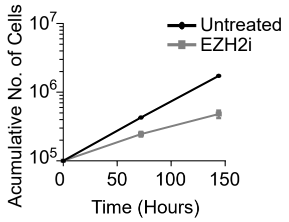

C

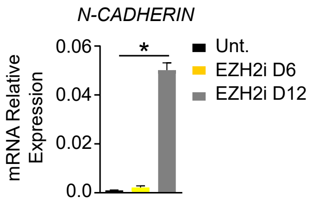

D

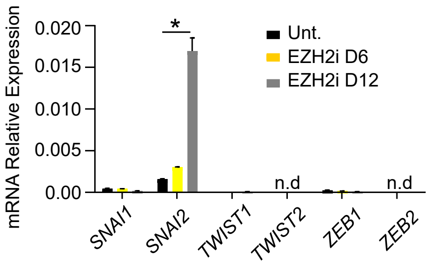

E

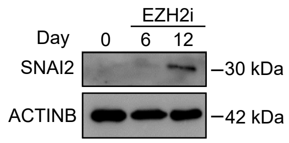

F

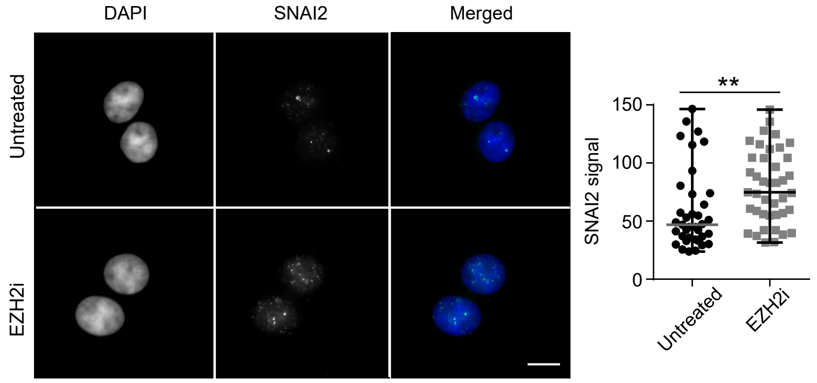

G

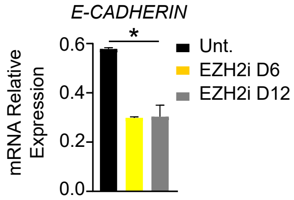

H

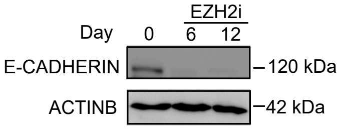

Supplement: Supplementary file 2 — Figure S1 [file 41419_2024_7011_MOESM2_ESM.pdf]
