# Supplementary figures and images for: EZH2 represses mesenchymal genes and upholds the epithelial state of breast carcinoma cells
Source: Cell Death Dis. 2024 Aug 22;15(8):609. doi: 10.1038/s41419-024-07011-y (PMC11341823; doi:10.1038/s41419-024-07011-y)

A

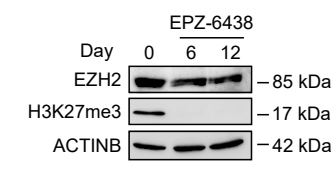

B

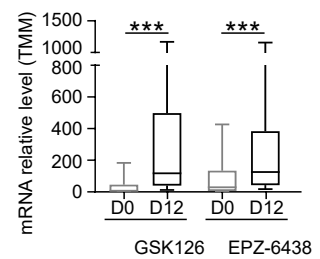

C

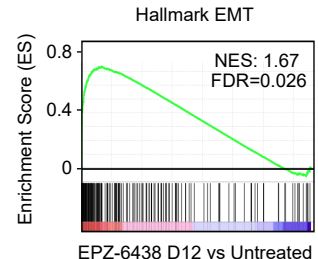

D

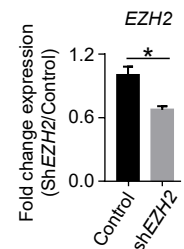

E

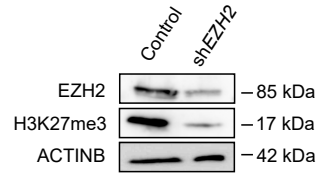

F

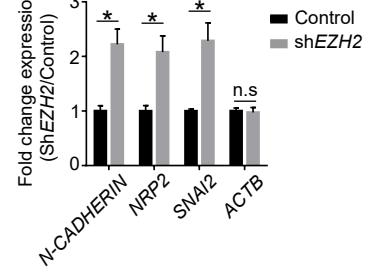

G

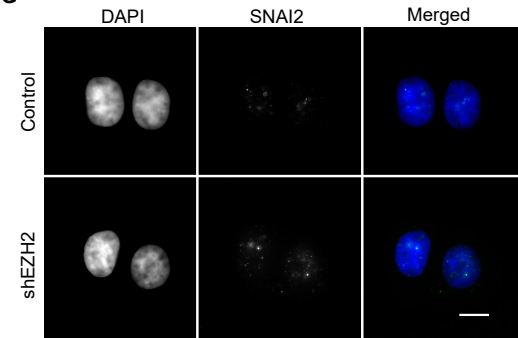

H

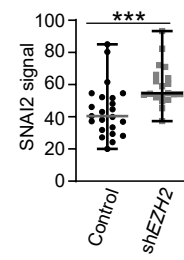

I

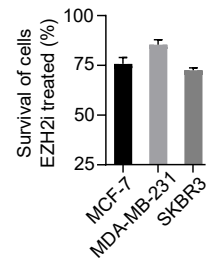

J

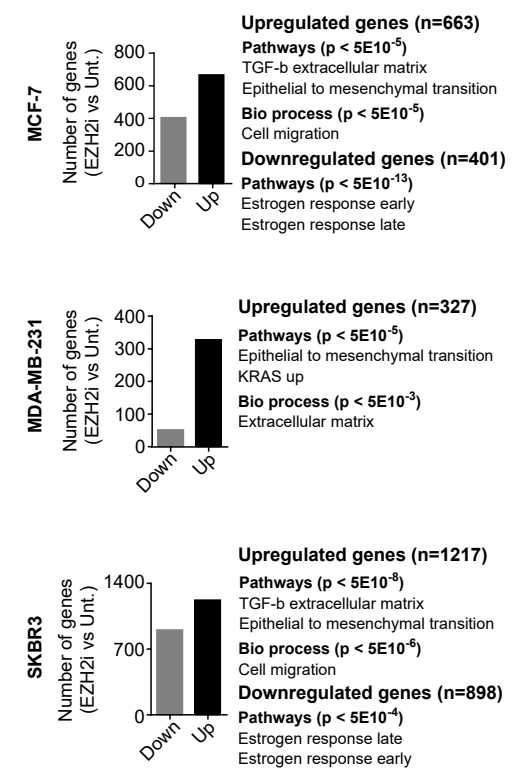

K

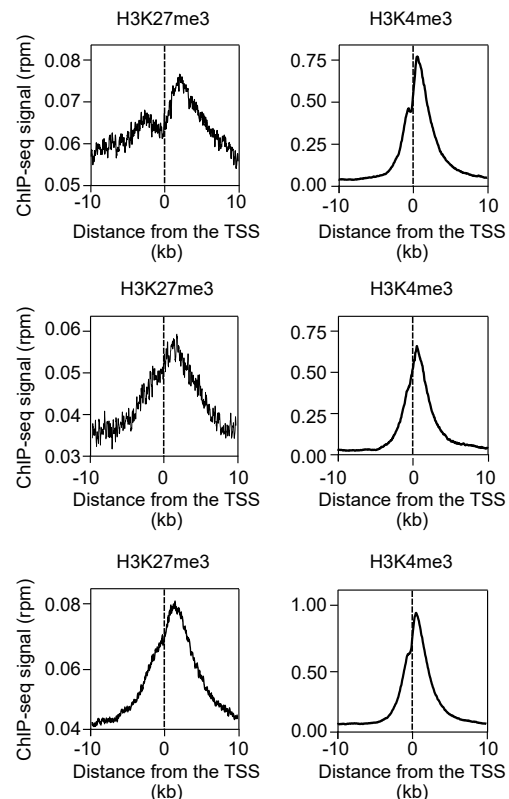

L

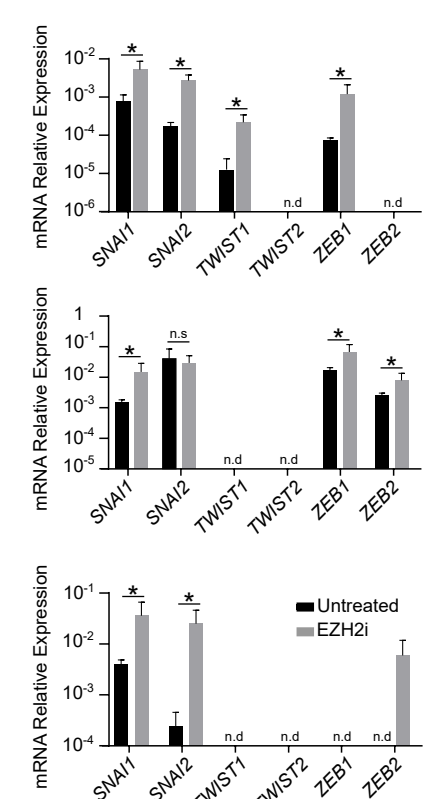

M

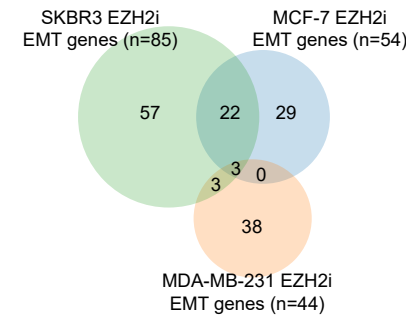

Supplement: Supplementary file 3 — Figure S2 [file 41419_2024_7011_MOESM3_ESM.pdf]

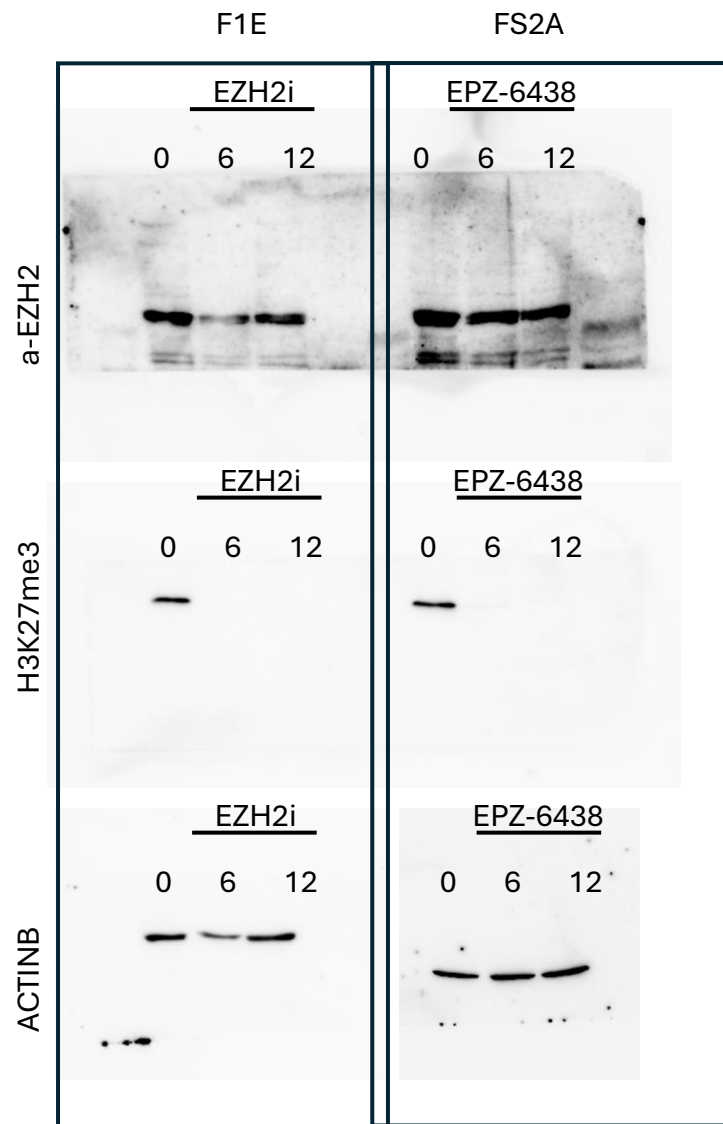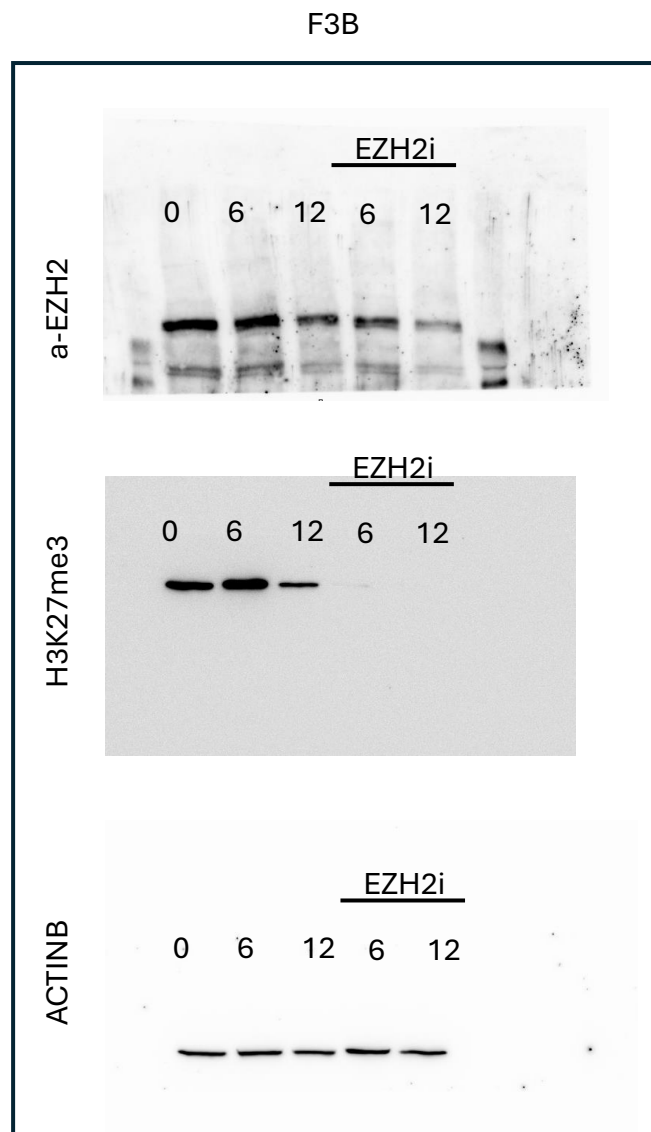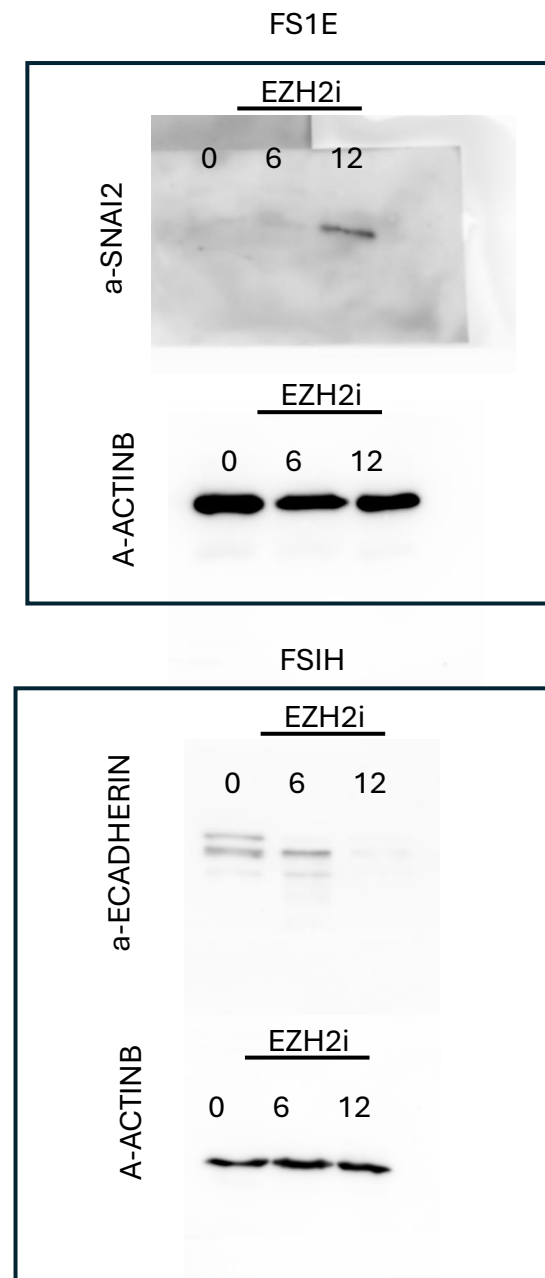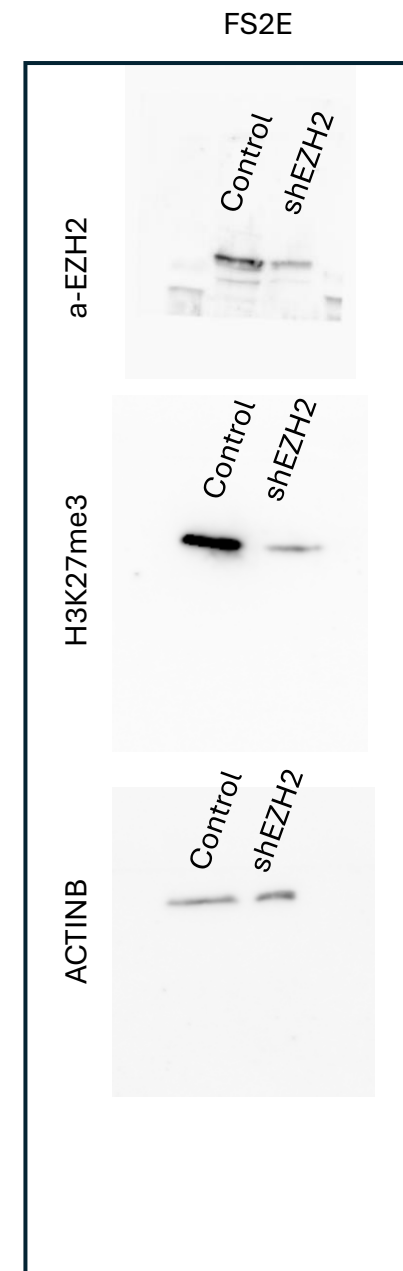

Supplement: Supplementary file 6 — original western blots images [file 41419_2024_7011_MOESM6_ESM.pdf]
